# The Accuracy of Video-Assisted Thoracic Surgery Pleural Biopsy in Patients with Suspected Diffuse Pleural Mesothelioma: A Real-Life Study

**DOI:** 10.3390/jcm15010042

**Published:** 2025-12-20

**Authors:** Ludovica Balsamo, Enrica Migliore, Eleonora Della Beffa, Luisa Delsedime, Paolo Olivo Lausi, Daniela Di Cuonzo, Filippo Lococo, Paraskevas Lyberis, Dario Mirabelli, Mauro Giulio Papotti, Enrico Ruffini, Francesco Guerrera

**Affiliations:** 1 Department of Cardio-Thoracic and Vascular Surgery, Azienda Ospedaliera Universitaria Città della Salute e della Scienza di Torino, 10126 Torino, Italy; ludovicabalsamo96@gmail.com (L.B.); paolo.lausi@unito.it (P.O.L.); p.lyberis@gmail.com (P.L.); enrico.ruffini@unito.it (E.R.); francesco.guerrera@unito.it (F.G.); 2 Department of Surgical Science, University of Torino, Corso Dogliotti, 14, 10126 Torino, Italy; 3 Unit of Cancer Epidemiology, Regional Operating Center of Piemonte (COR Piemonte), University of Torino and CPO-Piemonte, 10126 Torino, Italy; enrica.migliore@unito.it (E.M.); daniela.dicuonzo@unito.it (D.D.C.); 4 Department of Pathology, Azienda Ospedaliera Universitaria Città della Salute e della Scienza di Torino, 10126 Torino, Italy; luisa.delsedime@unito.it (L.D.); mauro.papotti@unito.it (M.G.P.); 5 Thoracic Surgery Unit, Università Cattolica del Sacro Cuore, 00168 Roma, Italy; filippo.lococo@policlinicogemelli.it; 6 Thoracic Surgery Unit, Fondazione Policlinico Universitario A. Gemelli IRCCS, 00168 Rome, Italy; 7 Department of Oncology, University of Torino, 10126 Torino, Italy

**Keywords:** malignant pleural mesothelioma, diffuse mesothelioma, diagnosis, thoracoscopy, pleural effusion, asbestos

## Abstract

**Background**: The heritage of occupational and environmental asbestos exposure in Piedmont, Italy, has resulted in an enduring diffuse pleural mesothelioma (DPM) epidemic. Our study aimed to investigate the accuracy of Pleural biopsy (PB) via thoracoscopy (or video-assisted thoracic surgery—VATS) and analyze the diagnostic path of patients who experienced an initial DPM misdiagnosis. **Methods**: Patients who underwent PB by VATS for suspected DPM from 2004 to 2013 were analyzed. The Registry of Malignant Mesothelioma (RMM) records were examined to cross-check incident cases and identify misdiagnosed DPM. The sensitivity and specificity of the initial PB assessment versus the final classification of cases by RMM were evaluated. **Results**: Data from 552 patients were analyzed, and DPM was diagnosed in 178 cases (32%). Sensitivity and specificity were 93% and 100%, respectively. The number of false-negative PBs was 14 (2%). Of those, 10 (71%) had an initial diagnosis of chronic pleuritis, 3 (28.5%) were initially classified as mesothelial proliferation, and 1 had reactive mesothelial proliferation. All of them reported a history of asbestos exposure, and the correct diagnosis was reached after a median of 160 days. One- and four-year survival rates were 52% and 10% in DPM PB-positive cases and 50% and 19% in false-negative cases. **Conclusions**: When a strong clinical suspicion after a negative PB remains, iterative biopsy attempts should be considered, especially if a history of asbestos exposure is reported. In high-volume centers, the DPM misdiagnosis rate remains low, and future advancements in diagnostic technologies could further increase the accuracy and efficacy of histologic diagnosis.

## 1. Introduction

Malignant pleural mesothelioma (DPM)—or diffuse pleural mesothelioma (DPM), as defined by the 2021 WHO classification—is an aggressive malignancy of the pleura, most associated with occupational or environmental asbestos exposure, and is characterized by poor prognosis and limited treatment options [1,2]. In the Piedmont region of Italy, a legacy of extensive asbestos use has resulted in one of the highest incidences of diffuse pleural mesothelioma in the country. To provide background context, Piedmont is in north-western Italy, covering approximately 25,400 km^2^ and with a population of about 4.3 million people. Historically, major industries have included automotive manufacturing, metallurgy, textiles, and asbestos-related manufacturing and processing, therefore increasing the incidence of pleural and peritoneal mesothelioma in the region.

The high mortality of DPM is a consequence of aggressive biological behavior and of limited and poorly effective treatment options [3,4].

Generally, clinical manifestations of mesothelioma are non-specific and include persistent cough, wheezing, and shortness of breath. The first radiologic exam is a chest x-ray showing a unilateral pleural effusion; in this clinical setting, it can be challenging to make an effective differential diagnosis between inflammatory pleural conditions, DPM, and pleural metastases from other malignancies [5,6,7].

In this context, the anamnestic data collection, especially exposure to asbestos or a history of previous cancer, and the clinical examination represent the cornerstone to guide the suspect and the diagnostic pathway for a correct DPM diagnosis. More than 90% of patients with pleural mesothelioma present with pleural effusion, and the association of pleural effusion and occupational asbestos exposure creates a strong clinical suspicion of mesothelioma. Radiologic imaging obtained with contrast-enhanced computed tomography (CT) and, when present, cytologic evaluation are often the first steps in diagnosing DPM [5,6]. Nevertheless, the sensitivity of the cytology analyzed is relatively low: cytologic examination findings are diagnostic in only 32% of patients. If pleural cytology is nondiagnostic in a patient with suspected mesothelioma, pleural biopsies are recommended [8,9]. Beyond clinical management, accurate diagnosis of DPM in Italy also has significant forensic and occupational health implications, influencing compensation claims and legal proceedings.

Thoracoscopy—either medical thoracoscopy or video-assisted thoracic surgery (VATS)—with targeted biopsies of the parietal pleura is considered the gold standard for histologic diagnosis, with sensitivity approaching 90% and a low complication rate [10,11,12]. Adequate tissue sampling enables precise histopathological subtyping into epithelioid, sarcomatoid, or biphasic variants, which carry prognostic and therapeutic implications [7,13]. The detailed subclassification and histological characterization of mesothelioma have prognostic and predictive implications for therapeutic approaches.

The present study aims to evaluate the diagnostic accuracy of VATS pleural biopsy (PB) in patients with suspected DPM, focusing on cases performed before the widespread adoption of molecular biomarkers. In addition, we analyze the diagnostic pathway of patients initially misdiagnosed, highlighting factors that may contribute to false-negative results.

## 2. Methods

### 2.1. Patients

**Figure 1:**
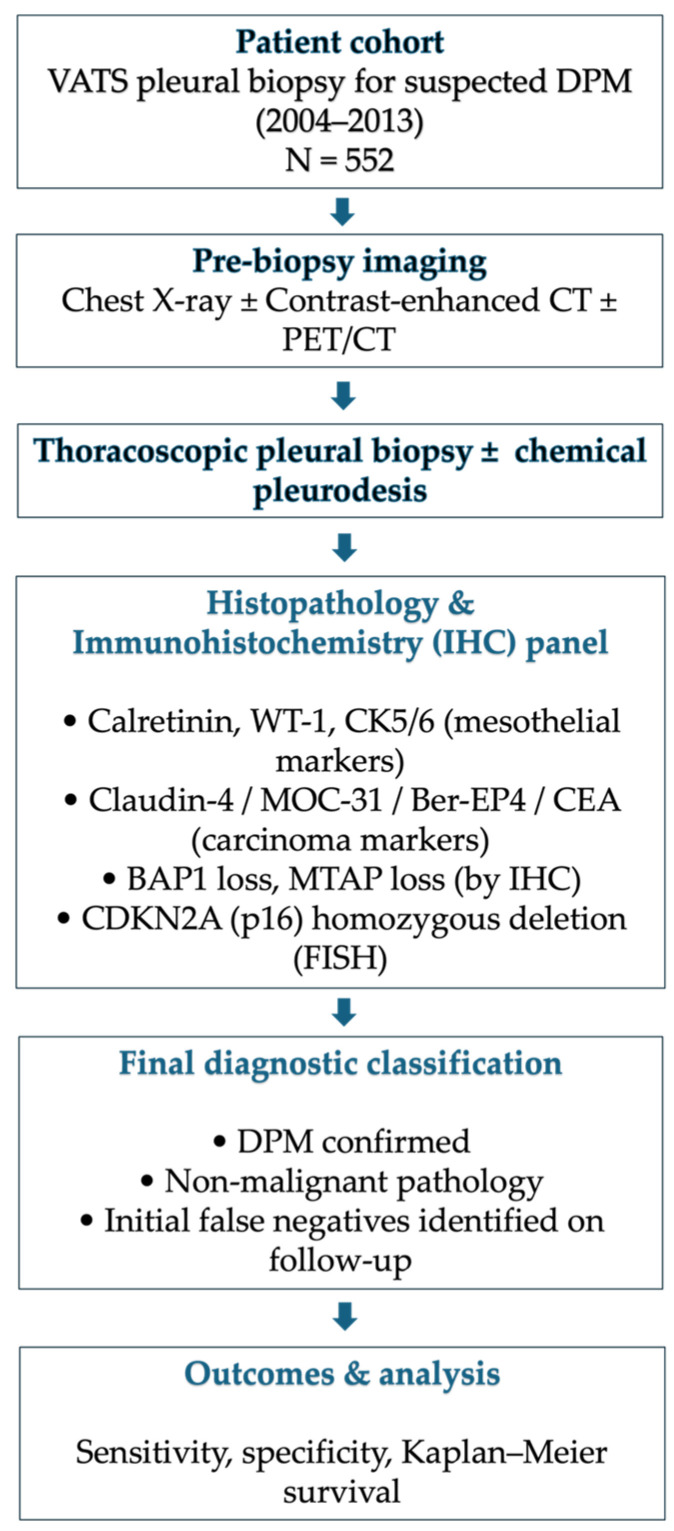
Patient’s management flowchart.

Patients were selected retrospectively from a consecutive cohort of individuals undergoing video-assisted thoracic surgery (VATS) for suspected diffuse pleural mesothelioma (DPM). The baseline clinical workup was performed according to international guidelines, including a physical examination, chest X-ray, computed tomography (CT) scans, and/or 18-fluorodeoxyglucose-positron emission tomography (PET) scans. Suspicion of DPM was based on clinical symptoms (e.g., unexplained pleural effusion, chest pain), radiological findings on CT and/or PET-CT suggestive of pleural malignancy, and occupational history of asbestos exposure when available (Figure 1). All consecutive eligible patients were included and cross-linked with the Piedmont Mesothelioma Registry to verify incident cases and final diagnoses. Data were collected from clinical, pathological, and surgical registries. The Piedmont Registry of Malignant Mesothelioma (RMM) records were examined to cross-check incident cases and identify misdiagnosed patients. Patients were included if they presented with clinical and/or radiologic suspicion of diffuse pleural mesothelioma (DPM), defined by one or more of the following: (i) unexplained unilateral pleural effusion or pleural thickening on chest X-ray or CT scan; (ii) pleural nodularity or metabolic activity on PET/CT suggestive of malignancy; (iii) documented history of occupational or environmental asbestos exposure associated with pleural disease; (iv) clinical symptoms compatible with pleural malignancy (e.g., chest pain, dyspnea) not explained by other causes. Exclusion criteria were (i) patients undergoing thoracoscopy for causes other than suspected mesothelioma (e.g., empyema, parapneumonic effusion, or metastatic pleural disease of known primary origin); (ii) patients with inadequate or missing histologic material; and (iii) patients with incomplete clinical or registry data preventing diagnostic verification by the Regional Malignant Mesothelioma Registry.

### 2.2. Ethical Aspects

The Registry of Malignant Mesothelioma (RMM) is a legally mandated, population-based registry established and regulated by Italian national legislation. The collection and processing of data within this registry are therefore carried out as an institutional public health duty and do not require prior approval by an Ethics Committee.

For the present study, we used anonymized data extracted from this legally mandated registry, and no additional procedures, interventions, or data collections beyond routine clinical practice were performed. All data were handled in accordance with Italian data protection laws and with the principles of the Declaration of Helsinki. No additional informed consent from patients was required. 

### 2.3. Study Outcomes

Clinical endpoints for this work were restricted to diagnostic performance; perioperative complications were not collected systematically because they were not predefined endpoints. Regarding missing data, endpoints were highly complete; gaps in descriptive covariates were limited, no imputation was performed, and subgroup comparisons were conducted as complete-case analyses. Reporting followed the STROBE recommendations.

### 2.4. Study Design and Definition

For this study, “misdiagnosis” was defined as an initial histopathological report that was discordant with the final confirmed diagnosis of DPM, as determined by the Piedmont Registry of Malignant Mesothelioma (RMM) after review of all available pathological, radiological, and clinical data. Cases were classified as false negatives when the initial VATS pleural biopsy failed to detect DPM. Still, subsequent diagnostic procedures (repeat biopsy, cytology, fine-needle aspiration, or open surgery) confirmed the diagnosis during follow-up.

This is an observational, monocentric retrospective study on prospectively recorded cases of patients who underwent pleural biopsy between January 2004 and December 2013. The study was conducted at the Thoracic Surgery Department of S. Giovanni Battista Hospital—Città della Salute e della Scienza di Torino.

The study period (January 2004–December 2013) was intentionally selected to capture a large, homogeneous cohort of patients treated before the widespread introduction of molecular and immunohistochemical biomarkers (e.g., BAP1, MTAP, CDKN2A) into routine diagnostic practice. After 2014, diagnostic workflows for pleural mesothelioma at our institution progressively incorporated molecular techniques, which would have introduced methodological heterogeneity and limited comparability across the study population. Moreover, our analysis aimed to assess the diagnostic accuracy of VATS pleural biopsy in the pre-molecular era, when histology alone was the cornerstone of diagnosis. This timeframe enabled us to provide a precise reference for centers where access to advanced molecular diagnostics remains limited.

### 2.5. Description of Procedures

Enrolled patients underwent a pleural biopsy via thoracoscopy using local anesthesia and deep sedation or general anesthesia, whichever anesthetic technique was decided after a multidisciplinary discussion including the thoracic surgeon and thoracic anesthesiologist.

As for surgical technique, a single 1 cm incision was made, and a rigid thoracoscope was inserted into the pleural cavity. The biopsy was performed on eye-visible lesions and on apparent normal pleura at random to increase diagnostic yield. At the end of the procedure, a 24 Fr chest tube was placed. In patients with diffuse, apparently neoplastic disease requiring symptom control, talc insufflation was performed as a palliative measure.

Concerning postoperative care, a chest X-ray was routinely performed on postoperative day 1 and after chest tube removal. The criteria for chest tube removal included a favorable chest X-ray, no air leak for at least 24 h, and overall fluid output not exceeding 250 mL/day. They drained fluid that was neither chylous nor hemorrhagic. Patients were discharged the day after chest tube removal if clinically stable.

### 2.6. Follow-Up

Vital status was confirmed for all patients as of 31 December 2023.

### 2.7. Statistical Analysis

Baseline patient characteristics are summarized as median and interquartile range (IQR) or number and percentage. Age distribution was assessed with the skewness–kurtosis test in Stata, confirming non-normality (Pr(skewness) < 0.001; joint *p* < 0.001), which was also evident in the Q–Q plot. Between-group comparisons used the Mann–Whitney U test for continuous variables and the chi-square or Fisher’s exact test for categorical variables. The sensitivity and specificity of the initial PB assessment versus the final classification of cases by RMM were evaluated. Overall survival (OS) was measured from the date of diagnosis to the date of death or last follow-up. Survival curves were estimated using the Kaplan–Meier method, and differences were evaluated using the log-rank test. Analyses were performed using Stata/MP, version 18.1 (StataCorp LLC., College Station, TX, USA). No formal a priori sample size calculation was performed due to the retrospective, real-life design. We included all consecutive eligible patients undergoing VATS pleural biopsy at our center between 2004 and 2013. Subgroup comparisons are reported with *p*-values as shown in Table 1 Survival analyses were conducted with a descriptive and exploratory intent. The study was not powered to detect prognostic differences, as survival was not a predefined endpoint of this diagnostic accuracy study.

## 3. Results

In the period of interest, 552 patients were treated. Most patients were male (69.9%), and the median age at surgery was 69.0 years (56.5–76.0). The demographic characteristics and clinical, radiologic, surgical, and pathologic features of these patients are reported in Table 2.

Overall, DPM was diagnosed in 178 cases (32%), and no false-positive PBs were observed. The Sensitivity and specificity were 93% and 100%, respectively. The number of false-negative PBs was 14 (2%). Of those, 10 (71%) were initially diagnosed with chronic pleuritis, 3 (28.5%) with atypical mesothelial proliferation, and 1 with reactive mesothelial proliferation. The specific details of patients who resulted in false-negative first-time biopsies are reported in Table 3. A history of asbestos exposure was reported in all false-negative PBs patients (14, 2%) and in 148 cases (83%) of actual positive patients. The most frequent histotype was epithelioid mesothelioma (72.5%).

In patients resulting as false negatives, the correct diagnosis was reached after a median of 160 days (interquartile range 86–243) as follows: 9 (64%) after a further PB by VATS, 3 (22%) by cytology examination of a pleural effusion, 1 (7%) by fine-needle biopsy and 1 (7%) by open surgery.

**Figure 2:**
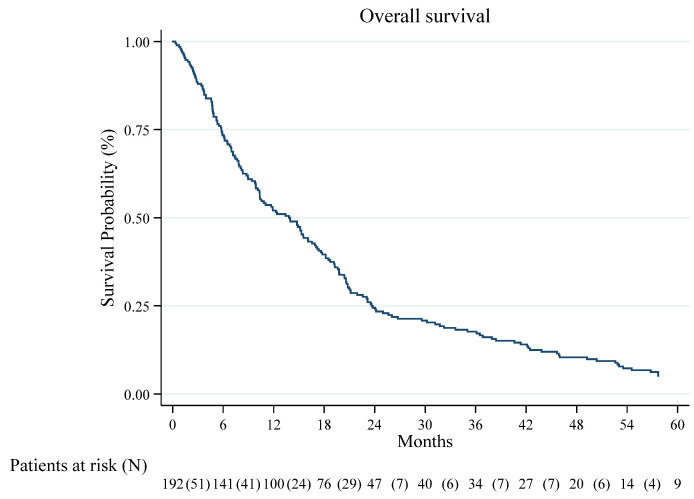
Kaplan–Meier Curve for Overall Survival.

**Figure 3:**
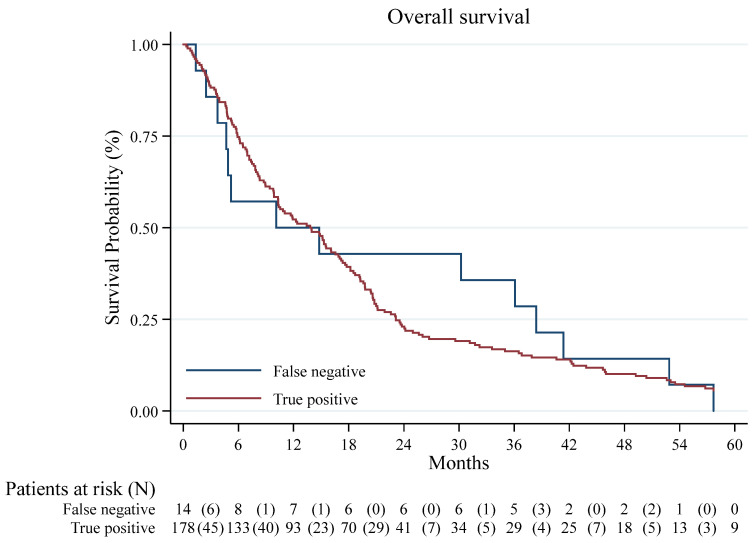
Kaplan–Meier curve for Survival: True positive vs. False Negative.

The median survival time of the patients with eventual DPM diagnosis was 13.8 months (CI 95%: 10.3–16.6). In the whole cohort, the 1- and 4-year OS were 52.1% (CI 95% 44.8–58.9) and 10.4% (CI 95% 6.6–15.2), respectively (Figure 2). The 1- and 4-year survival was 52.2% (CI 95% 44.7–59.3) and 10.1% (CI 95% 6.2–15.1) in DPM PB positive cases and 50.0% (CI 95% 22.9–72.2) and 14.3% (CI 95% 2.3–36.6) in false-negative cases (log-rank test *p* = 0.813), respectively (Figure 3).

## 4. Discussion

The study was conducted on a cohort of 552 consecutive patients who underwent VATS pleural biopsies between 2004 and 2013. The results indicate that (I) the sensitivity and specificity of pleural biopsies via VATS for DPM were elevated (93% and 100%, respectively); (II) the majority of false negative (FN) biopsies required further pathological tissue sampling during the patient disease history to achieve an adequate diagnosis of mesothelioma (or malignant pleural disease in few cases reported in Table 3 where the diagnosis was not conclusive for the specific mesothelioma histotype); (III) the overall survival resulted in being comparable between false negative (FN) and true positive (TP), probably undelaying the poor prognosis that these patients unfortunately share and the few therapeutic options available.

Because pleural effusion is usually the first clinical finding in DPM, thoracentesis with cytology is often the first invasive diagnostic procedure performed. However, pleural effusion cytology has a low diagnostic sensitivity (32–51.3%) and is inadequate to identify different mesothelioma histotypes [3]. Currently, the introduction of Biomarkers such as Loss of BAP1 or MTAP expression (by immunohistochemistry) or homozygous deletion of CDKN2A (by fluorescence in situ hybridization) has improved the sensitivity of cytological examination. Still, histological examination remains the diagnostic gold standard in pleural malignancies.

The current clinical practice at our center for diagnosing mesothelioma focuses on using a panel of at least two mesothelial markers and two carcinoma markers to achieve optimal diagnostic accuracy. Commonly employed positive mesothelial markers include calretinin, Wilms tumor-1 (WT-1), and cytokeratin 5/6 (CK5/6), which typically show intense nuclear or cytoplasmic staining in mesothelial cells [8]. Negative carcinoma markers such as claudin-4, MOC-31, Ber-EP4, and CEA are used to exclude metastatic adenocarcinoma, particularly lung or breast carcinoma. In recent years, ancillary markers such as BRCA1-associated protein 1 (BAP1) loss by nuclear staining and methylthioadenosine phosphorylase (MTAP) loss [9,10] have emerged as highly specific for malignant mesothelial proliferation, aiding in the distinction from benign mesothelial hyperplasia. Additionally, detection of a homozygous CDKN2A deletion by fluorescence in situ hybridization (FISH) can further support the diagnosis in challenging cases [11].

As a direct consequence of the need to analyze pleural tissue, video-assisted thoracic surgery is the most reliable diagnostic tool for direct visualization of the pleural cavity and for obtaining high-quality biopsies [4]. An adequate tissue specimen will enable us to distinguish histologic subtypes of diffuse pleural mesothelioma and perform molecular testing, which influences prognosis and treatment [3]. For this reason, surgical diagnosis represents a primary and central step in the whole process of disease management [14].

According to various authors, the number of required biopsies to achieve an accurate diagnostic yield has been reported to range from 5 to 10 samples, and it is strongly advised to obtain repeated pleural biopsies at the same site if adhesions and fibrotic tissue are visible on the thoracoscopic view. Concurrently, the quality of pleural biopsies can be improved by adequate instrumentation: a rigid or semi-flexible thoracoscope, combined with a diathermy knife or cryobiopsy technique [15], might ensure a better diagnostic yield and, therefore, a conclusive diagnosis.

Furthermore, obtaining a definitive diagnosis and determining whether a biopsy specimen is benign or malignant can present some additional issues to address: the distinction between mesothelial hyperplasia and epithelioid mesothelioma, and the distinction between reactive pleural fibrosis and sarcomatoid or desmoplastic mesothelioma might be complicated; this is the situation in which immunohistochemistry is most helpful [16].

The diagnosis of “chronic pleuritis” cannot be attributed to a specific etiology; mesothelioma can be paucicellular and may be difficult to distinguish from reactive fibrinous pleuritis, especially if the pleura shows thickening or adhesions [15]. Because of the benign course in 85% of patients with non-specific pleuritis, the standard approach is to wait and see and to repeat biopsies only in a limited group of patients, when signs of the disease appear to be stronger [15]. Of course, this “wait and watch” approach needs to account for later diagnosis and patients accessing treatments with advanced-stage disease. Trying to mitigate this situation, in our study, biopsy was repeated in a short time in patients whose first pathological report was identifying the disease as chronic pleuritis if they reportedly had any clinical history of asbestos exposure and suggestive radiological findings, and 71% of patients with an initial diagnosis of chronic pleuritis were diagnosed as mesothelioma.

On the other hand, the term “atypical mesothelial hyperplasia” indicates a proliferating mesothelium with some but not all the histological or cytological features of well-differentiated mesothelioma [17] and encompasses benign mesothelial hyperplasia, premalignant mesothelial proliferation, in situ mesothelioma, and early well-differentiated malignant mesothelioma. In diagnosing atypical mesothelial hyperplasia, the question is whether it’s better to wait and see, as some authors suggest [18], or to repeat an invasive diagnostic procedure sooner to ensure a prompt diagnosis [5].

In our study, the indications for a repeated pleural biopsy in this group of patients were thoracic pain, recurrent pleural effusion, and radiologic findings suggestive of malignant pleural disease. The results of our series show that 28.5% of patients with an initial diagnosis of atypical mesothelial hyperplasia were diagnosed with mesothelioma.

False-negative patients were identified as those with an initial VATS pleural biopsy that failed to detect DPM. Still, subsequent diagnostic procedures (repeat biopsy, cytology, fine-needle aspiration, or open surgery) confirmed the diagnosis during follow-up.

In the literature, the false-negative rate in diagnosing mesothelioma after thoracoscopic biopsy ranges from 5% to 25% [19]. In our study, the false-negative rate was significantly lower than reported in the literature, around 2%. Of those, 71% had an initial diagnosis of chronic pleuritis, 28.5% had atypical mesothelial hyperplasia, and only 1 case had reactive mesothelial proliferation. All the patients presenting with an inconclusive diagnosis were followed up in time until a second suspicion of advancing malignant pleural disease. They were again biopsied, obtaining at this point a definitive diagnosis. The correct diagnosis was reached after a median of 160 days: 64% after a further pleural biopsy via video-assisted thoracic surgery, 22% by cytology of a pleural effusion, 7% by fine-needle biopsy, and 7% by open surgery. According to other authors, the negative patients should be followed for a minimum of one year to allow for timely detection of occult pleural malignancy [6,7,8,9,10,11,12,13,14,15,16,17,18,19,20]. Our clinical routine is to monitor patients not diagnosed with mesothelioma for at least 24 months.

In our cohort, overall survival was comparable between false negatives and true positives. Interestingly, a small subset of patients demonstrated survival exceeding five years despite a diagnosis of malignant pleural mesothelioma, a finding that warrants consideration given the typically poor prognosis of this disease. Several factors may account for this observation. First, these patients were predominantly affected by the epithelioid histologic subtype, which is well recognized to confer a more indolent disease course and a more prolonged median survival than the biphasic or sarcomatoid variants. Second, earlier-stage detection in some cases likely enabled access to potentially curative interventions, such as pleurectomy/decortication or extrapleural pneumonectomy, in combination with adjuvant chemotherapy or radiotherapy. Referral to and management within a high-volume tertiary center may have further contributed to these outcomes by facilitating timely diagnosis, multidisciplinary decision-making, and optimized perioperative care. Finally, we cannot exclude the influence of individual patient factors, such as preserved performance status and limited comorbidity burden, which are known to impact treatment tolerance and prognosis. Kaplan–Meier curves and log-rank tests were used for descriptive purposes; we did not fit Cox proportional hazards models or report hazard ratios because survival was not a predefined endpoint of this diagnostic accuracy study, and the size of the false-negative subgroup (N = 14) precluded stable time-to-event modeling. Survival outcomes in our cohort were analyzed exploratorily to provide contextual information; however, the study design and sample size were not intended or powered for prognostic inference.

The debate over the timing of re-biopsy remains open and challenging, as it involves balancing patient safety, the risk of complications, and the need for a definitive diagnosis to adequately assess treatment or palliate disease symptoms through talc insufflation. The implications of such diagnostic delays are profound. Early-stage diagnosis is crucial for offering potentially curative interventions such as pleurectomy/decortication combined with multimodal therapy. Furthermore, systemic treatments such as chemotherapy and newer immunotherapies are more effective when the tumor burden is lower, and the patient’s performance status is better. Nowadays, new technologies can help surgeons select more effective areas for diagnosis [13]. Additionally, integrating emerging techniques such as circulating tumor DNA analysis and novel immunohistochemical markers holds promise for reducing false negatives and better stratifying patients for appropriate therapies earlier, potentially improving survival outcomes and quality of life in this difficult-to-treat cancer [21].

In recent years, liquid biopsy has emerged as a promising, minimally invasive diagnostic tool that could complement or, in some cases, replace repeated tissue biopsies. Studies have identified specific biomarkers, such as miR-126, miR-625-3p [22,23,24,25,26,27,28], and SMRP (soluble mesothelin-related peptides), which show diagnostic and prognostic relevance in mesothelioma [7,16,29,30,31]. Moreover, liquid biopsy could provide critical insights in cases with inconclusive histology or insufficient tissue, especially when repeat thoracoscopic procedures pose a high risk. This diagnostic approach is not yet available in our center. As such, its use remains for the moment confined to research settings or specialized institutions.

Our study presents some limitations that should be considered when interpreting the results. Firstly, the monocentric, retrospective design inherently limits the generalizability of the findings, as the data were collected from a single center and reflect the clinical practices, patient population, and diagnostic strategies unique to that institution, thereby introducing selection and spectrum/referral bias. Cross-linkage with the regional mesothelioma registry mitigates case loss and misclassification of outcomes. As for multivariable adjustment for confounders, we deliberately did not fit regression models because the number of events (false negatives) was small (N = 14), which would yield unstable, overfit estimates and potentially misleading inference. We therefore present unadjusted comparisons and explicitly frame them as exploratory.

Additionally, the thoracoscopic procedures analyzed in this study were performed on patients at various stages of disease progression, introducing heterogeneity in clinical presentation, disease burden, and biopsy complexity. Furthermore, these procedures were performed by different surgeons with varying levels of expertise and clinical experience, potentially introducing subjectivity and inter-operator variability.

Despite these limitations, the strength of our study lies in the rigorous follow-up of patients through a dedicated Rare Malignant Mesothelioma (RMM) program, which ensured high completeness and consistency in longitudinal data collection. This structured follow-up enhances the reliability of our conclusions and supports the robustness of our diagnostic assessment.

## 5. Conclusions

In conclusion, our study’s findings emphasize that, when clinical suspicion of diffuse pleural mesothelioma (DPM) persists despite a negative pleural biopsy, further diagnostic evaluation, including iterative biopsy attempts, should be strongly considered. This is particularly relevant in patients with a documented history of asbestos exposure, who remain at high risk for DPM. Moreover, our data confirm that in high-volume tertiary referral centers, the rate of DPM misdiagnosis remains relatively low, suggesting that experience and multidisciplinary collaboration contribute significantly to diagnostic accuracy. Looking ahead, continued advancements in imaging modalities, molecular diagnostics, and minimally invasive biopsy techniques hold the promise of further improving early detection and diagnostic precision in pleural malignancies.

## Figures and Tables

**Table 1:** Characteristics of DPM Patients.

	DPM Cases (N = 192)		
	True Positives (N = 178)	False Negatives (N = 14)	*p*-Value
Sex			
Male	128 (71.9)	8 (57.1)	0.239
Female	50 (28.1)	6 (42.9)	
Age (median, IQR)	73.0 (68.0–77.0)	71.0 (61.0–77.0)	0.230
History of asbestos exposure			
No	18 (10.1)	-	0.408
Yes	148 (83.2)	14 (100)	
N/A	12 (6.7)	-	
Histology			
Biphasic	31 (17.4)	-	0.022
Epithelioid	129 (72.5)	9 (64.3)	
Not specified	6 (3.4)	2 (14.3)	
Sarcomatoid	10 (5.6)	3 (21.4)	

**Table 2:** Baseline Characteristics in the overall population.

Total	(N = 552)
Age	
Median (Q1, Q3)	69.0 (56.5, 76.0)
Min, Max	17.0, 91.0
Gender	
Males	386 (69.9%)
Females	166 (30.1%)
History of asbestos exposure	
No	221 (40.0%)
Yes	319 (57.8%)
N/A	12 (2.2%)
Histology	
Biphasic	31 (16.3%)
Epithelioid	138 (72.6%)
Not specified	8 (4.2%)
Sarcomatoid	13 (6.8%)

**Table 3:** Characteristics of cases with initial negative findings after biopsies in thoracoscopy.

Patient ID	Gender	Age	Initial Diagnosis Date	Initial Diagnosis	Final Diagnosis	Basis for Final Diagnosis	Additional Findings Leading to Diagnosis	Days to Final Diagnosis	Death
10	F	58	March 2004	Nonspecific fibrosing pleuritis	Epithelioid mesothelioma	Positive cytology on pleural effusion	Chest CT showing diffused pleural thickening and infiltration of sub-pleural and mediastinal adipose tissue	196	March 2007
14	F	61	September 2004	Inflammatory mesothelial hyperplasia	Epithelioid mesothelioma	Repeated biopsy during a second thoracoscopy		119	February 2005
5	M	73	March 2006	Nonspecific fibrosing pleuritis	Sarcomatoid mesothelioma	Repeated biopsy during a second thoracoscopy	Chest CT scan showing diffused pleural thickening	85	August 2006
12	F	67	November 2005	Nonspecific fibrosing pleuritis	Sarcomatoid mesothelioma	Repeated biopsy during a second thoracoscopy		188	October 2006
15	F	68	December 2006	Atypical mesothelial hyperplasia	Sarcomatoid mesothelioma	Repeated biopsy during a second thoracoscopy		21	June 2007
7	M	77	March 2009	Nonspecific fibrosing pleuritis	Pleural neoplasm	Positive cytology on pleural effusion	Chest CT scan showing right sided pleural thickening	283	April 2010
16	F	53	January 2011	Atypical mesothelial hyperplasia	Epithelioid mesothelioma	Repeated biopsy during a second thoracoscopy	PET scan showing diffused enhancement of the left mediastinal and parietal pleura	86	February 2012
11	M	80	October 2010	Nonspecific fibrosing pleuritis	Pleural neoplasm	Repeated fine needle biopsy	PET scan showing diffused enhancement of the right parietal pleural and pleural thickening	120	February 2014
13	M	73	February 2011	Nonspecific fibrosing pleuritis	Epithelioid mesothelioma	Repeated biopsy during a second thoracoscopy		208	February 2015
4	M	73	May 2011	Atypical mesothelial hyperplasia	Epithelioid mesothelioma	Repeated biopsy during a second thoracoscopy	PET scan showing diffused enhancement of the left parietal pleura and pleural thickening	133	February 2016
8	M	69	July 2011	Nonspecific fibrosing pleuritis	Epithelioid mesothelioma	Repeated biopsy during a second thoracoscopy		243	December 2016
2	M	78	January 2013	Nonspecific fibrosing pleuritis	Epithelioid mesothelioma	Repeated fine needle biopsy	Chest CT showing lump-shaped right pleural thickening and chest wall infiltration with right chest bulging	65	May 2016
6	F	77	May 2013	Nonspecific fibrosing pleuritis	Epithelioid mesothelioma	Repeated biopsy during a second thoracoscopy	Chest CT showing diffused pleural thickening	520	January 2016
18	M	48	April 2007	Nonspecific fibrosing pleuritis	Epithelioid mesothelioma	Surgical excision	Chest CT (2009) showing multiple pleural nodular thickenings; PET scan (2012) showing a large expanding lesion in the right parietal pleura	1940	January 2013

## Data Availability

The data that support the findings of this study are derived from the institutional surgical and pathological registries and the Piedmont Regional Malignant Mesothelioma Registry (RMM). In accordance with Italian data protection laws and institutional policy, the raw data are not publicly available due to privacy and ethical restrictions. Anonymized datasets used and analyzed during the current study are available from the corresponding author upon reasonable request and subject to approval by the institutional RMM (https://www.cpo.it/en/data/rmm#schede (accessed on 16 December 2025).

## Abbreviations

DPM	Diffuse Pleural Mesothelioma
VATS	Video-Assisted Thoracic Surgery
PB	Pleural Biopsy
RMM	Regional Malignant Mesothelioma Registry (Piedmont Registry)
WHO	World Health Organization
CT	Computed Tomography
PET	Positron Emission Tomography
PET-CT	Positron Emission Tomography–Computed Tomography
OS	Overall Survival
FN	False Negative
TP	True Positive
IQR	Interquartile Range
CI	Confidence Interval
STROBE	Strengthening the Reporting of Observational Studies in Epidemiology
FISH	Fluorescence In Situ Hybridization
IHC	Immunohistochemistry
BAP1	BRCA1-Associated Protein 1
MTAP	Methylthioadenosine Phosphorylase
CDKN2A	Cyclin Dependent Kinase Inhibitor 2A
WT-1	Wilms Tumor 1
CK5/6	Cytokeratin 5/6
MOC-31	Anti-epithelial related antigen
BRCA 1	Breast Related Cancer Antigen 1
CEA	Carcinoembryonic Antigen
SMRP	Soluble Mesothelin-Related Peptides
ctDNA	Circulating Tumor DNA
miRNA	MicroRNA
